# Polyphenols and Health Benefits: 2nd Edition

**DOI:** 10.3390/foods14244340

**Published:** 2025-12-17

**Authors:** Nayeli Edith Navarro García, Joyce Trujillo, Victoria Ramírez

**Affiliations:** 1 División de Materiales Avanzados, Instituto Potosino de Investigación Científica y Tecnológica (DMA-IPICYT), San Luis Potosí 78216, Mexico; nayeli.navarro@ipicyt.edu.mx; 2 Secretaría de Ciencia, Humanidades, Tecnología e Innovación (SECIHTI), Benito Juárez, Ciudad de México 03940, Mexico; 3 Departamento de Nutrición Animal, Instituto Nacional de Ciencias Médicas y Nutrición Salvador Zubirán, Tlalpan, Ciudad de México 14080, Mexico

Polyphenols are naturally occurring bioactive secondary metabolites found in medicinal plants, vegetables, grains, seeds, fruits, beverages, and other foods [1]. For example, wild fruits and berries are excellent sources of polyphenols [2]. The latter are a chemical family of compounds comprising several subclasses: phenolic acids, stilbenes, tannins, coumarins, and flavonoids [2]. The last ones have potential health benefits and may be non-essential nutrients derived from their therapeutic effects on chronic diseases [1,3]. These flavonoids consist of two aromatic rings connected by a three-carbon bridge [2]. Depending on the quantity and arrangement of their hydroxyl groups, alkylation and glycosylation, they primarily exhibit antioxidant and radical scavenging properties. Furthermore, they have demonstrated the ability to balance oxidative stress, exhibit antimicrobial properties, and reduce the effects of metabolic diseases and cancer (antiproliferative properties).

In this regard, this special issue, “Polyphenols and Health Benefits, Volume II,” presents six research articles and one review. These publications highlight the importance of a rich biological source of polyphenols and antioxidants, which can contribute to beneficial effects (antioxidant, anti-inflammatory, antiproliferative, antihypertensive, antimicrobial, and metabolic regulation). The articles showed that polyphenols are used in various diseases, including cancer, cardiovascular, metabolic, infectious, and neurodegenerative diseases, highlighting their potential as alternative or complementary therapeutic approaches. Of note, this second volume explores the polyphenol content of cloves, aronia, green tea, seed extracts, cacao, pitaya, and other polyphenol-rich fruits and plants. These manuscripts describe the main functional and bioactive metabolites associated with polyphenols and validate their functional activities. The results presented could predicted developments of encapsulated extracts, functional juices or foods, supplements, and computational tools to design bioactive-rich foods with potential applications in the prevention and co-treatment of chronic diseases.

Red fruit pomace is valued for its polyphenols, vitamins, dietary fiber, low calories, and health benefits [4]. In Contribution 1, it was demonstrated that chokeberry and blueberry fruits, which are characteristic of cold climates and contain high amounts of polyphenols, are derived from anthocyanins, procyanidins, and hydroxycinnamic acids (Contribution 5). Therefore, there are potential formulations for the design of functional foods and nutraceuticals. The highest polyphenols percentage was found in chokeberry extracts through a simple and feasible process. Both exhibited antioxidant, anti-inflammatory, and antiproliferative properties in cell culture–based colorectal cancer studies, which is mainly attributed to the presence of polyphenols and anthocyanins. It’s very interesting that these bioactive compounds can control the tumor microenvironment; the effects appeared to be mediated through pathways involving Erk signaling and Akt-1 inhibition, pathways that regulate proliferation and cell survival. Future research should examine how extracts of blueberry and chokeberry pomace affect adhesion and migration in colorectal cancer cells. Colon cancer is considered one of the top three causes of death. Hence, the importance of continuing to study beneficial strategies for its treatment.

Perilla frutescens seeds, grown primarily in Thailand, showing that their extract exhibits antioxidant, anti-inflammatory, anticancer, antiallergic, antibacterial, and antifungal activity; restores the intestinal microbiota; controls blood insulin; protects against oxidative stress; and can also induce iron chelation. Contribution 2 showed by HPLC content of rosmarinic acid, apigenin, luteolin, and quercetin in the extract of these seeds, which are related to tumor reduction in colorectal cancer cell lines. In this study, reported the existence of antioxidant activity derived from a DPPH^•^ radical scavenging assay. Chromatin condensation and DNA fragmentation were also achieved in HT-29 cells with treatments of 50 µg/mL (0.85 µg/mL of rosmarinic acid 390 and 0.08 µg/mL of luteolin) of the extract for 24 h, exhibiting the inhibition of tumor progression, since programmed cell death is promoted, through modulation of the G1 cell cycle, proteins or mitochondrial signaling (cytochrome C and caspase-3).

This extract is also capable of suppressing proinflammatory cytokines, inhibiting kappa-B (signaling pathway) and protein kinase, key regulators of inflammatory responses. Suggesting that the perilla frutescens functions as a natural agent in the prevention and treatment of colorectal cancer. Nevertheless, future studies reporting the use of this extract in dietary supplements are expected to determine its efficacy and safety in vivo testing.

Furthermore, there is a close relationship between cancer and the intestinal microbiome, and natural polyphenols (e.g., resveratrol and curcumin), along with probiotics and prebiotics, show a potential role in modulating immune responses and enhancing chemotherapy [5]. Thus, exhibits molecular interactions with the human diet, and implementing several emerging therapies could be through this route. Concerning this, in contribution 3, the proposal utilizes dietary polyphenols, derived from their bioactive potential in colon diseases. In general, probiotics and prebiotics promote the beneficial activity of the microbiome. The preference is to use diets rich in polyphenols, since these are starting to be considered as prebiotics due to their ability to improve inflammatory bowel disease, irritable bowel syndrome, and colon cancer. Its main mechanism of action is through the modulation and strengthening of the intestinal barrier. This has potential application in the prevention of gastrointestinal diseases and cancer. Another advantage of implementing diets rich in polyphenols is that reduces dysbiosis, limiting the proliferation of harmful bacterial genera associated with the presence of cancer. That is to say, due to the increased proliferation of Bifidobacterium and Lactobacillus and the decreased presence of taxa (Clostridium and Escherichia coli). Also, this review shows that resveratrol exhibits antiproliferative activity by inducing apoptosis and regulating cell proliferation through the induction of p53. Phenolic bioactive compounds regulate the cell cycle and positively activate caspase 3.

Besides, there exist chemical carcinogens that can enter the human body due to interactions with the environment or with certain foods. Some of the most common are aflatoxin B1 (present in Aspergillus molds), ethylene oxide (used in sterilizing medical instruments), chloroethylene oxide (used in agriculture), carbamate epoxide (a byproduct of fermentation), glycidamide (fried foods), styrene oxide (plastics), propylene oxide (refrigerants), 2-cyanoethylene oxide (adhesives), and beta-propiolactone (viral vaccines treatments). All these pro-carcinogenic forms are metabolized by Cytochrome P450, and the reactive metabolites interact with DNA, causing mutations that initiate cancer through chain reactions. Contribution 4 shows how these chemicals react through interactions between nitrogen atoms and guanine. The flavonoids used as chemopreventive agents are most commonly those high in catechins, such as green tea and cocoa. Nevertheless, the mechanisms of action by which the interaction between flavonoids and chemical agents induces the chemoprotective effect are not yet understood.

In this contribution, a quantum mechanical model was designed in which various forms of polyphenols were subjected to physiological conditions in an attempt to interpret how carcinogen elimination occurs. This was determined by measuring the activation energies involved in alkylation processes. Guanine was used as a model structure. Fourteen density functionals were also employed.

According to the results, the best interactions were obtained by Density Functional Theory due to the meta-approximation exchange-correlation functionals for generalized gradients of type M11-L and MN12-L. The information provides specific data on reaction mechanisms, possible state configurations, transition states, and alkylation kinetics between phenolic compounds and epoxy carcinogens. This allows the identification of the polyphenols that participate most efficiently in the depuration of carcinogens.

Moreover, other uses of polyphenols as a dietary strategy to serve as adjuvant treatments for chronic diseases, such as blood pressure control, diabetes, and other chronic diseases. In this context, another interesting contribution, number 5, is that they specifically refer to the use of *Aronia melanocarpa* (chokeberry), previously mentioned fruit. Because studies on the metabolic effects that these polyphenols can have on humans are few, they decided to carry out a feasibility clinical trial in healthy adults, which would allow them to determine changes in fecal microbiota and possible improvements in metabolomic profiles. Briefly, by administering 100 mL of chokeberry juice for 30 days, they were able to demonstrate favorable control of total cholesterol levels. A reduction in blood glucose levels was observed even when high-fat foods were consumed. In this case, this is related to adequate inhibition of the enzyme dipeptidyl peptidase IV, which regulates epithelial glucose transport. Regarding the inflammatory response, the chokeberry juice consumption did not induce changes; a longer experimental period is possibly required. To metabolic responses, this consumption induces changes in asparagine and tyrosine serum levels, both essential amino acids for the proper functioning of metabolic processes, indicating that consumption of this polyphenol-rich juice is adequate in modulating these serum amino acids, optimizing health. Based on this contribution, different clinical experiments could be designed to maximize the doses used to obtain greater benefits.

Another interesting food rich in polyphenols in this Special Issue was clove *(Syzygium aromaticum*)*,* which is a primary bioactive compound, eugenol. Still, it has disadvantages such as intense flavor, instability, and poor solubility. The relevant contribution in this sixth number is the use of encapsulant agents, such as gum Arabic and maltodextrin, plant-derived polysaccharides, which preserve functionality, bioactivity, and promote the release of the bioactive compounds. Both encapsulants are soluble and have low viscosity. A eugenol content of 87.7 mg/g was determined in the clove extract. Several formulations were created with varying amounts of gum Arabic and maltodextrin, ranging up to 0:100 ratios. It was determined that the use of gum Arabic increases wet control, which can contribute to premature release and degradation of the compound. Using maltodextrin decreases this content, as does the density, although a higher value is preferable for storage efficiency. Loading efficiencies exceeding 70% were achieved in terms of total phenols and eugenol. New chemical bonds were not formed, suggesting that the chemical properties of the biocomposite remained unchanged regardless of the encapsulation ratios used. This study proposes encapsulation as an efficient method for preserving bioactive components. Furthermore, the bioavailability of polyphenols in the intestinal tract was improved, promoting this method’s potential application in functional foods.

Another disease with a high incidence worldwide is high blood pressure, for which conventional treatments can present side effects or induce resistance in patients. This is why there is interest in using biomolecules present in traditional medicine, and functional foods are arising. Among the possible adjuvant treatments is pitaya juice, a fruit predominant in desert areas of Mexico, which has a high content of betalains and phenolic compounds. In general, the mechanisms involved in blood pressure regulation include the nervous system, the hormonal system, and vasodilatory molecules (like NO, prostaglandins, and endothelin). This study (Contribution 7) focused on voltage-gated Ca^2+^ channels, since the contraction capacity of blood vessels depends on them.

A food of interest more for its chemical composition, highlighting its polyphenol content among others, its study is observed in the Contribution 7, where the vasodilatory effect was determined from a concentrated pitaya juice, a cactus fruit (*Stenocereus huastecorum*). Using an ex vivo and in vivo model, the first are rat thoracic aortic rings, which showed a maximum relaxation of 66% after exposure to a concentrated pitaya juice. It was established that this effect is primarily due to the blockade of Ca^2+^ channels and is independent of the endothelium and K^+^ channels. To complement these results, an evaluation was conducted in a hypertension experimental model in rats, where a dose of 400 mg/mL of concentrated pitaya juice achieved a hypotensive effect (20%) within the first hour, generating normotensive blood pressure levels for a period of 72 h. Furthermore, the juice concentrate was fractionated to obtain the bioactive compounds responsible for vasorelaxation, and it was suggested that these compounds are of a glycoside nature and could be a complex mixture of disaccharides, dimeric disaccharides, or even tetrasaccharides, since the presence of two monosaccharide units was observed. This gives both the concentrated juice and its fraction potential therapeutic applications in the control of arterial hypertension.

As we observed here, all these manuscripts demonstrated that polyphenols or their metabolites have essential biological actions as antioxidants or regulators of lipid and carbohydrate metabolism, which may have a direct impact on non-communicable diseases, including obesity, metabolic syndrome, diabetes, and even cancer, as well as their comorbidities.

## Data Availability

The data described here are publicly available in the special issue Polyphenols and Health Benefits: Volume II.
